# Complete genome anatomy of the emerging potato pathogen *Dickeya solani* type strain IPO 2222^T^

**DOI:** 10.1186/s40793-016-0208-0

**Published:** 2016-11-29

**Authors:** Slimane Khayi, Pauline Blin, Teik Min Chong, Kok-Gan Chan, Denis Faure

**Affiliations:** 1 Institute for Integrative Biology of the Cell (I2BC), CNRS CEA Univ. Paris-Sud, Université Paris-Saclay, Avenue de la Terrasse, 91198 Gif-sur-Yvette cedex, France; 2 Division of Genetics and Molecular Biology, Institute of Biological Sciences, Faculty of Science, University of Malaya, 50603 Kuala Lumpur, Malaysia

**Keywords:** Short genome report, *Dickeya solani*, Blackleg, Soft rot, Genome, Potato

## Abstract

Several species of the genus *Dickeya* provoke soft rot and blackleg diseases on a wide range of plants and crops. *Dickeya solani* has been identified as the causative agent of diseases outbreaks on potato culture in Europe for the last decade. Here, we report the complete genome of the *D. solani* IPO 2222^T^. Using PacBio and Illumina technologies, a unique circular chromosome of 4,919,833 bp was assembled. The G + C content reaches 56% and the genomic sequence contains 4,059 predicted proteins. The ANI values calculated for *D. solani* IPO 2222^T^ vs. other available *D. solani* genomes was over 99.9% indicating a high genetic homogeneity within *D. solani* species.

**Electronic supplementary material:**

The online version of this article (doi:10.1186/s40793-016-0208-0) contains supplementary material, which is available to authorized users.

## Introduction

*Dickeya* are pectinolytic enterobacteria that cause soft rot and blackleg diseases on a wide range of crops worldwide including potato plants (*Solanum tuberosum*) [1, 2]. They are equipped with an arsenal of plant-cell wall degrading enzymes that macerate tuber and stem tissues provoking disease symptoms [3]. In the beginning of the 2000′s, *D. solani* emerged as a novel species causing blackleg and soft rot diseases on potato in Europe and Mediterranean Basin [4]. Initially, several pectinolytic strains isolated from potatoes grown in Europe and Israel, were identified as members of the *Dickeya* genus, but shown to exhibit distinctive genetic and physiological traits (biovar 3). Thereafter, additional phylogenetic and biochemical analyses have brought these isolates into a distinct clade called *D. solani* [5–8]. The *D. solani* strain IPO 2222 ^T^ was isolated from infected potato plants in The Netherlands in 2007 [9].

To date, 12 draft genomes of *D. solani* are available in GenBank databases. Among them, the genome of the strain IPO 2222 ^T^ was sequenced using 454-pyrosequencing with a low average genome coverage (14×). The resulting draft genome is composed of 91 contigs that were assembled in a single scaffold [9]. In this report, we combined Illumina and Pacific Biosciences technologies to provide a complete genome sequence of the strain IPO 2222 ^T^. We also highlighted some phylogenetic and phenotypic key-features of the *D. solani* species.

## Organism information

### Classification and features

**Fig. 1:**
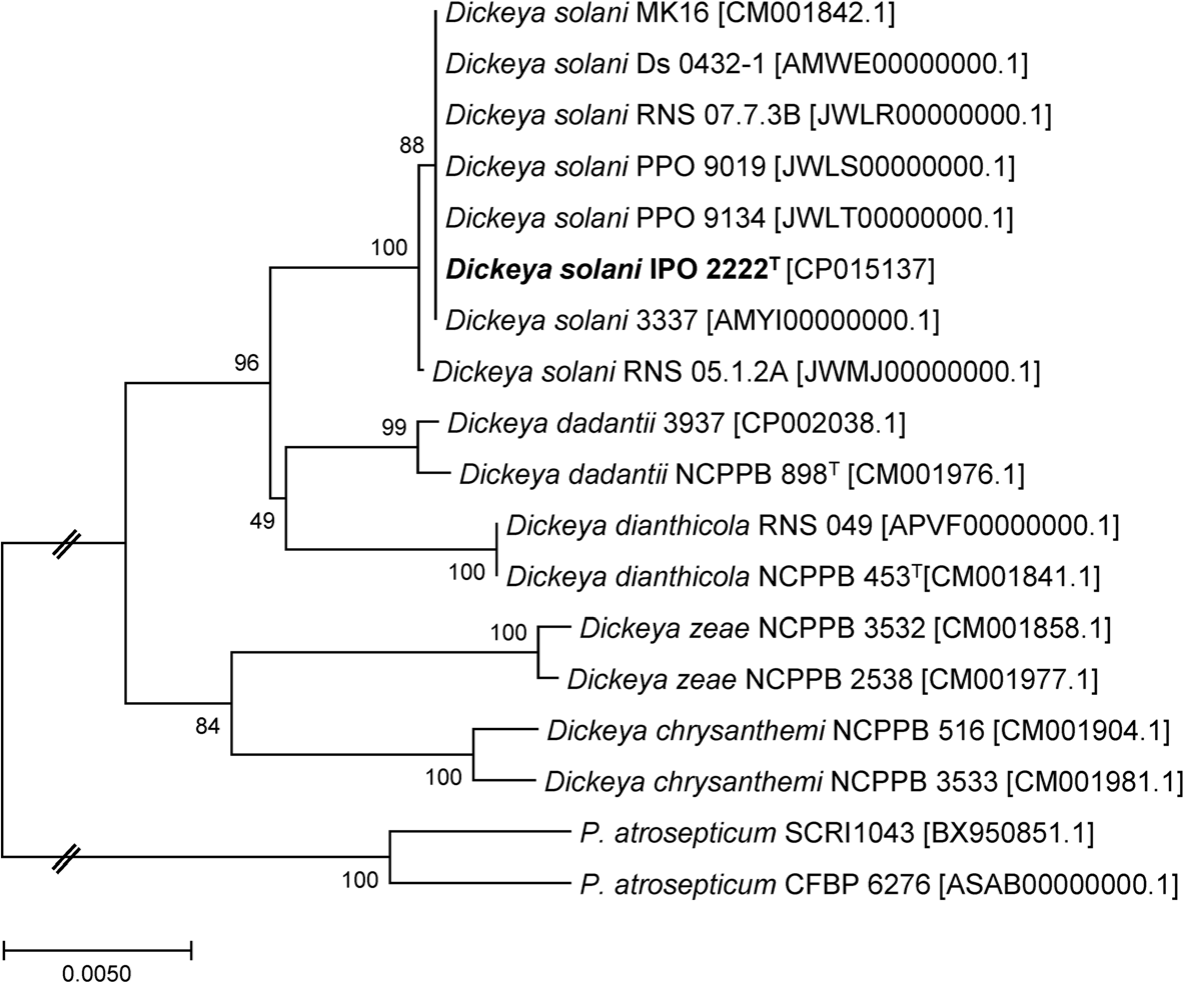
Phylogenetic tree highlighting the relative position of *D. solani* IPO 2222^T^ within other *Dickeya* and *Pectobacterium* species. The unique *gapA* gene was retrieved from each of the complete and draft genomes that are available in NCBI database; alignment was generated using MUSCLE [23]; the evolutionary history was inferred using the Neighbor-Joining method [24] and the evolutionary distances were computed using the Maximum Composite Likelihood method [25]. Phylogenetic analyses were conducted using MEGA7 software [26]

*D. solani* IPO 2222 ^T^ belongs to the order of *Enterobacteria* and the class of *Gammaproteobacteria*. The *gapA*-based phylogenetic tree (Fig. 1) was congruent with the previously reported trees inferred from MLSA [8, 10], gathering all *D. solani* strains in a distinct clade within the *Dickeya* genus. The *gapA* housekeeping gene was chosen instead of 16S rRNA gene because the sequence analysis of *gapA* permit a highly resolved view of distinction between members of the *Dickeya* genus [8, 10].

**Fig. 2:**
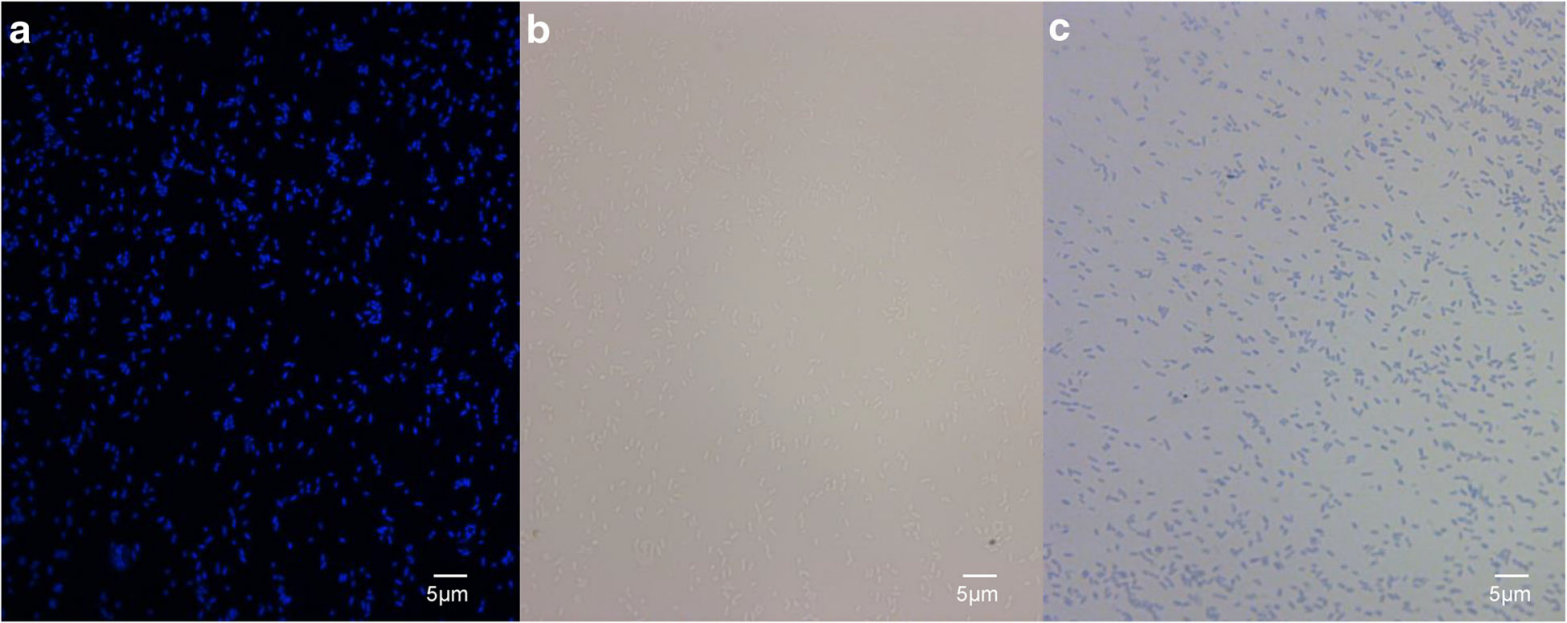
Photomicrographs of *D. solani* IPO 2222^T^ using DAPI (4′,6-diamidino-2-phenylindole) staining (**a**), differential interference contrast (**b**) and blue methylene staining (**c**). These photomicrographs show the rod shaped forms of *D. solani* species

*D. solani* IPO 2222 ^T^ is a Gram negative, non-spore-forming, motile and facultative anaerobic bacterium with rod shaped cells (0.9x2.0 μm) (Fig. 2) [8]. The strain IPO 2222 ^T^ grows in TY medium (tryptone 5 g/L, yeast extract 3 g/L and agar 1.5%) at 28 °C forming 1–2 mm colonies within 24 h. It produces phosphatase and indole and belongs to *Dickeya* biovar 3 as described previously [10]. Distinctive metabolic abilities of *D. solani* species were described using BIOLOG system [11]; among them, *D. solani* IPO 2222 ^T^ uses urea as sole nitrogen source (Additional file 1: Figure S1). *D. solani* IPO 2222 ^T^ was recovered form naturally infected potato plants showing blackleg and soft rot symptoms. Its aggressiveness was confirmed by infecting potato tubers and plants in greenhouse assays (Additional file 2: Figure S2). In addition, its ability to colonize the roots and stem tissues and to provoke disease symptoms has been reported using green fluorescent protein-tagged strain [12].

The strain IPO 2222 ^T^ has been registered at the Belgian Co-ordinated Collections of Micro-organisms (LMG 25993 ^T^), the National Collection of Plant Pathogenic Bacteria in UK (NCPPB 4479 ^T^), and the International Center for Microbial Resources - French collection of plant-associated bacteria (CFBP 8199 ^T^). MIGS of *D. solani* strain IPO 2222 ^T^ is summarized in Table 1.

**Table 1:** Classification and general features of *Dickeya solani* strain IPO 2222^T^ [13]

MIGS ID	Property	Term	Evidence code^a^
	Classification	Domain *Bacteria*	TAS [15]
		Phylum *Proteobacteria*	TAS [27]
		Class *Gammaproteobacteria*	TAS [28, 29]
		Order “*Enterobacteriales”*	TAS [28, 29]
		Family *Enterobacteriaceae*	TAS [30]
		Genus *Dickeya*	TAS [1]
		Species *Dickeya solani*	TAS [8]
		Type strain: IPO 2222^T^ (CP015137)	
	Gram stain	negative	TAS [8]
	Cell shape	Rod	TAS [8]
	Motility	Motile	IDA
	Sporulation	Non sporulating	NAS [8]
	Temperature range	Mesophilic	TAS [8]
	Optimum temperature	39°C	TAS [8]
	pH range; Optimum	Not reported;7	IDA
	Carbon source	D-Arabinose, Mannitol	TAS [8]
MIGS-6	Habitat	Rhizosphere	TAS [8]
MIGS-6.3	Salinity	0.5% NaCl (w/v)	TAS [31]
MIGS-22	Oxygen requirement	Facultatively anaerobic	TAS [8]
MIGS-15	Biotic relationship	free-living	TAS [8]
MIGS-14	Pathogenicity	Pathogenic	NAS [8]
MIGS-4	Geographic location	Netherlands	TAS [8, 9]
MIGS-5	Sample collection	2007	TAS [8, 9]
MIGS-4.1	Latitude	Not reported	NAS
MIGS-4.2	Longitude	Not reported	NAS
MIGS-4.4	Altitude	Not reported	NAS

^a^Evidence codes - IDA: Inferred from Direct Assay; TAS: Traceable Author Statement (i.e., a direct report exists in the literature); NAS: Non-traceable Author Statement (i.e., not directly observed for the living, isolated sample, but based on a generally accepted property for the species, or anecdotal evidence). These evidence codes are from the Gene Ontology project [32]

## Genome sequencing information

### Genome project history

The genome sequence of *D. solani* strain IPO 2222 ^T^ was sequenced using two technologies, PacBio RSII and Illumina NextSeq 500. This organism was selected based on the agricultural relevance as an emerging pathogen with a significant impact on the potato production and trade in Europe and around the world. Project information is available from Genome Online database number Gp0138842 under the Gold study number Gs0118682 at Joint Genome Institute. The complete genome sequence is also deposited in GenBank under the accession number CP015137. In Table 2, we provide a summary of the project information and its association with MIGS [13].

**Table 2:** Project information

MIGS ID	Property	Term
MIGS 31	Finishing quality	Complete genome
MIGS-28	Libraries used	Paired-end
MIGS 29	Sequencing platforms	Illumina NextSeq500, PacBio
MIGS 31.2	Fold coverage	450X
MIGS 30	Assemblers	CLC Genomics
MIGS 32	Gene calling method	NCBI Prokaryotic Genome Annotation Pipeline
	Locus Tag	A4U42
	Genbank ID	CP015137
	GenBank Date of Release	16 Mai 2016
	GOLD ID	Gp0138842
	BIOPROJECT	PRJNA317288
MIGS 13	Source Material Identifier	IPO 2222^T^
	Project relevance	Agricultural

### Growth conditions and genomic DNA preparation

*D. solani* IPO 2222 ^T^ was routinely cultured in TY medium at 28 °C. Genomic DNA extraction was performed from 5 mL overnight culture using a phenol-chloroform purification method followed by an ethanol precipitation as described by Wilson [14]. Quantification and quality control of the DNA was completed using a NanoDrop (ND 1000) device, Qubit® 2.0 fluorometer and agarose (1.0%) gel electrophoresis.

### Genome sequencing and assembly

Second generation sequencing was performed using NextSeq 500 (Illumina, CA, USA) at the I2BC platform (Gif-sur-Yvette, France). A paired-end library was constructed with an insert size of 390 bp and sequencing was carried out using 2 × 151 bp paired-end read module. The *de novo* assembly (length fraction, 0.5; similarity, 0.8) was performed using CLC Genomics Workbench (v8.0) software (CLC Inc, Aarhus, Denmark). After quality (quality score threshold 0.05) and length (above 40 nucleotides) trimming of the sequences, 33 contigs (N50 = 266,602 bp) were generated (CLC parameters: automatic determination of the word and bubble sizes with no scaffolding) with a 450× average genome coverage. The largest contig length was 617,431 bp.

Third generation sequencing was performed using PacBio RSII (Pacific Biosciences, CA, USA) at the University of Malaya (Kuala Lumpur, Malaysia). The SMRTbell template library at the size of 20 kbp was constructed using the commercial Template Preparation Kit (Pacific Biosciences, CA, USA) followed by sequencing using P6/C4 sequencing chemistry with sequence collection time set at 240 min. Prior to assembly, short reads (less than 500 bp) were filtered off and the minimum polymerase read quality used for mapping of sub-reads from a single zero-mode waveguides was set at 0.75. In total 146,263 reads were obtained (N50 value was 9,161 bp) and total base pair number was at 1,070,191,526 resulting in a 217× average genome coverage. Reads were assembled using RS_HGAP_Assembly software (V2.0). The cut-off length of seeding reads was set at 13,304 bp in order to serve as a reference for the recruitment of shorter reads for preassembly. The resulted consensus accuracy based on multiple sequence alignment of the sub-reads was at 99.99%.

The *de novo* Illumina-contigs were used to verify the RS_HGAP assembly by blasting them against the PacBio sequence. In addition, the trimmed Illumina reads were mapped (length fraction, 0.5; similarity, 0.8) against the PacBio sequence and errors (SNPs and InDels), that might be generated by homopolymers during PacBio sequencing, were searched and corrected using basic variant calling tool from CLC genomic workbench. Using these two sets of sequences, the complete genome sequence was approved and circularized.

### Genome annotation

The complete genome of *D. solani* IPO 2222 ^T^ was annotated using the NCBI prokaryotic genome annotation pipeline [15]. The protein coding gene prediction process begin by an alignment using ProSplign [16] where only complete alignments with 100% identity to a reference protein are kept for final annotation. Then the remaining frameshift or partial alignments were further analyzed by GeneMarkS+ [17]. To identify structural rRNA, the pipeline uses BLASTn search against the curated reference set. tRNAscan-SE was used to identify the tRNAs [18]. The CRISPRs are identified by using the CRISPR database [15].

## Genome properties

**Fig. 3:**
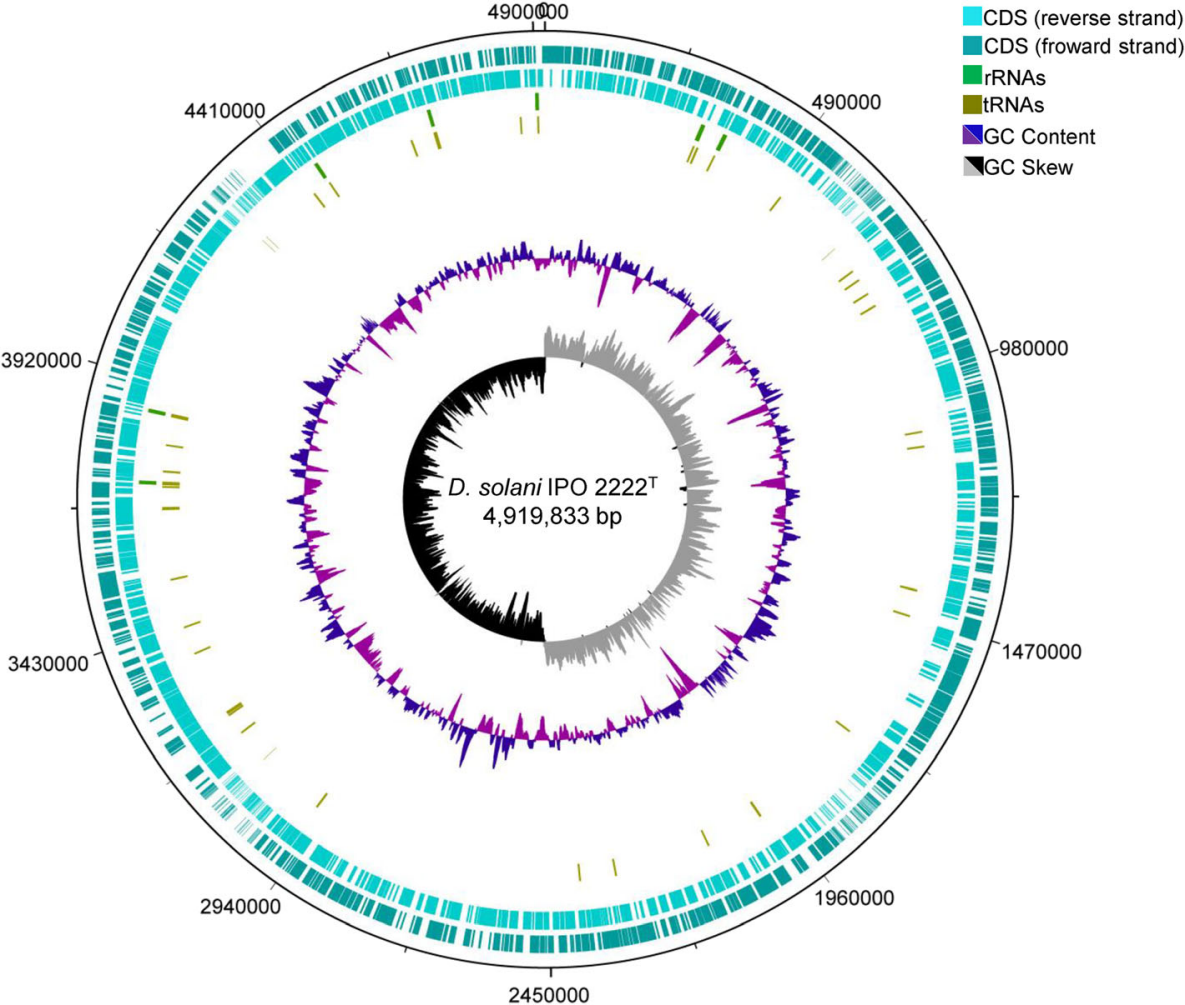
Graphical circular map of *D. solani* IPO 2222^T^ chromosome

The detailed information about *Dickeya solani* IPO 2222 ^T^ genome is provided in Table 3. The genome is constituted of one circular chromosome, 4,919,833 bp in size. The annotation predicted 4,208 genes including 4,059 CDSs (Table 4), 104 RNA genes (75 tRNA, 22 rRNA and 7 ncRNA genes) and 45 pseudo genes. The G + C reached 56%. The graphical genome map is provided in the Fig. 3.

**Table 3:** Genome statistics

Attribute	Value	% of total
Genome size (bp)	4,919,833	100.00
DNA coding (bp)	4,243,944	86.33
DNA G + C (bp)	2,767,155	56.24
DNA scaffolds	1	100.00
Total genes	4,208	100.00
Protein coding genes	4,104	97.5
RNA genes	104	2.5
Pseudo genes	45	1.06
Genes in internal clusters	1,093	25.97
Genes with function prediction	3,670	87.21
Genes assigned to COGs	3,365	79.97
Genes with Pfam domains	3,788	99.02
Genes with signal peptides	386	9.17
Genes with transmembrane helices	953	22.65
CRISPR repeats	1	-

**Table 4:** Number of genes associated with general COG functional categories

Code	Value	% age^a^	Description
J	234	6.09	Translation, ribosomal structure and biogenesis
A	1	0.03	RNA processing and modification
K	305	7.94	Transcription
L	112	2.92	Replication, recombination and repair
B	0	0.00	Chromatin structure and dynamics
D	42	1.09	Cell cycle control, Cell division, chromosome partitioning
V	90	2.34	Defense mechanisms
T	216	5.62	Signal transduction mechanisms
M	245	6.38	Cell wall/membrane biogenesis
N	106	2.76	Cell motility
U	82	2.14	Intracellular trafficking and secretion
O	138	3.59	Posttranslational modification, protein turnover, chaperones
C	222	5.78	Energy production and conversion
G	324	8.44	Carbohydrate transport and metabolism
E	438	11.41	Amino acid transport and metabolism
F	96	2.5	Nucleotide transport and metabolism
H	192	5.0	Coenzyme transport and metabolism
I	130	3.39	Lipid transport and metabolism
P	281	7.32	Inorganic ion transport and metabolism
Q	93	2.42	Secondary metabolites biosynthesis, transport and catabolism
R	282	7.34	General function prediction only
S	175	4.56	Function unknown
-	843	20.03	Not in COGs
^b^Total	4,683	120	

^a^The percentage is based on the total number of protein coding genes in the annotated genome

^b^The total does not correspond to 4,208 CDS because some genes are associated with more than one COG functional categories

## Insights from the genome sequence

*D. solani* species is genetically highly homogenous with 99.9% in genomic similarity (ANI value) [19, 20]. Between two given *D. solani* genomes, the number of variations (SNPs/InDels) is below one hundred. For example, when *D. solani* strain 3337 and *D. solani* strain IPO 2222 ^T^ were compared, 49 variations were observed: 15 were located out of CDS and 34 within CDS [19]. Only a few of *D. solani* genomes (strains RNS 07.7.3B, PPO 9019 and PPO 9134) exhibited a higher number of variations (>1000) because they acquired *D. dianthicola* genes by horizontal gene transfer [19]. None horizontal gene transfer from *D. dianthicola* was observed in strain IPO 2222 ^T^.

Plant-cell wall degrading enzymes comprising pectinases, proteinases and cellulases, play a major role in the plant tissue maceration process [21]. Indeed, 10 pectates lyase enzymes (genes *pelABCDEILXWZ*) were predicted in strain IPO 2222 ^T^ genome; they showed a 93.3% average nucleotide identity when compared to the orthologous genes of *D. dadantii* 3937.

Recent comparative analyses underlined the major genetic and metabolic divergences between *Dickeya solani* species and the nearest clades that are *D. dandatii* (ANI 94%) and *D. dianthicola* (ANI 92%) [11, 19]. *D. solani* is characterized by a low content of phages elements and CRISPR system: in strain IPO 2222 ^T^ genome, only one CRISPR cluster (208 bp) was identified. Using PHAST tool [22], the strain IPO 2222 ^T^ harbors one questionable prophage (11 CDSs) in a 10,687 bp region. In addition, some genomic regions were shown to be specific for *D. solani* species and contain some metabolic and NRPS/PKS encoding genes [11].

## Conclusions

The complete sequence of *D. solani* IPO 2222 ^T^ is the first complete genome of a member of this species, the type strain. This work provides a substantial resource in terms of knowledge of the bacterial genetic material. It may help to understand the successful fitness of *D. solani* in invading potato fields, opening the way to new control strategies against this phytopathogen.

## Additional files

Additional file 1: Figure S1. Growth curves of *D. solani* IPO 2222^T^, *D. dadantii* 3937 and *D. dianthicola* RNS 049 in the presence of urea as a sole nitrogen source. Data were collected from duplicates. (TIFF 296 kb)
Additional file 2: Figure S2. Symptoms of *D. solani* IPO 2222^T^ on potato plants (a) and tubers (b). (TIFF 8904 kb)

## Abbreviations

CDS: Coding DNA sequence
CRISPR: Clustered regularly interspaced short palindromic repeats
MIGS: Minimum information on the genome sequence
MLSA: Multi-locus sequence analysis
