# Supplementary figures and images for: Complete genome anatomy of the emerging potato pathogen Dickeya solani type strain IPO 2222T
Source: Stand Genomic Sci. 2016 Nov 29;11:87. doi: 10.1186/s40793-016-0208-0 (PMC5127095; doi:10.1186/s40793-016-0208-0)

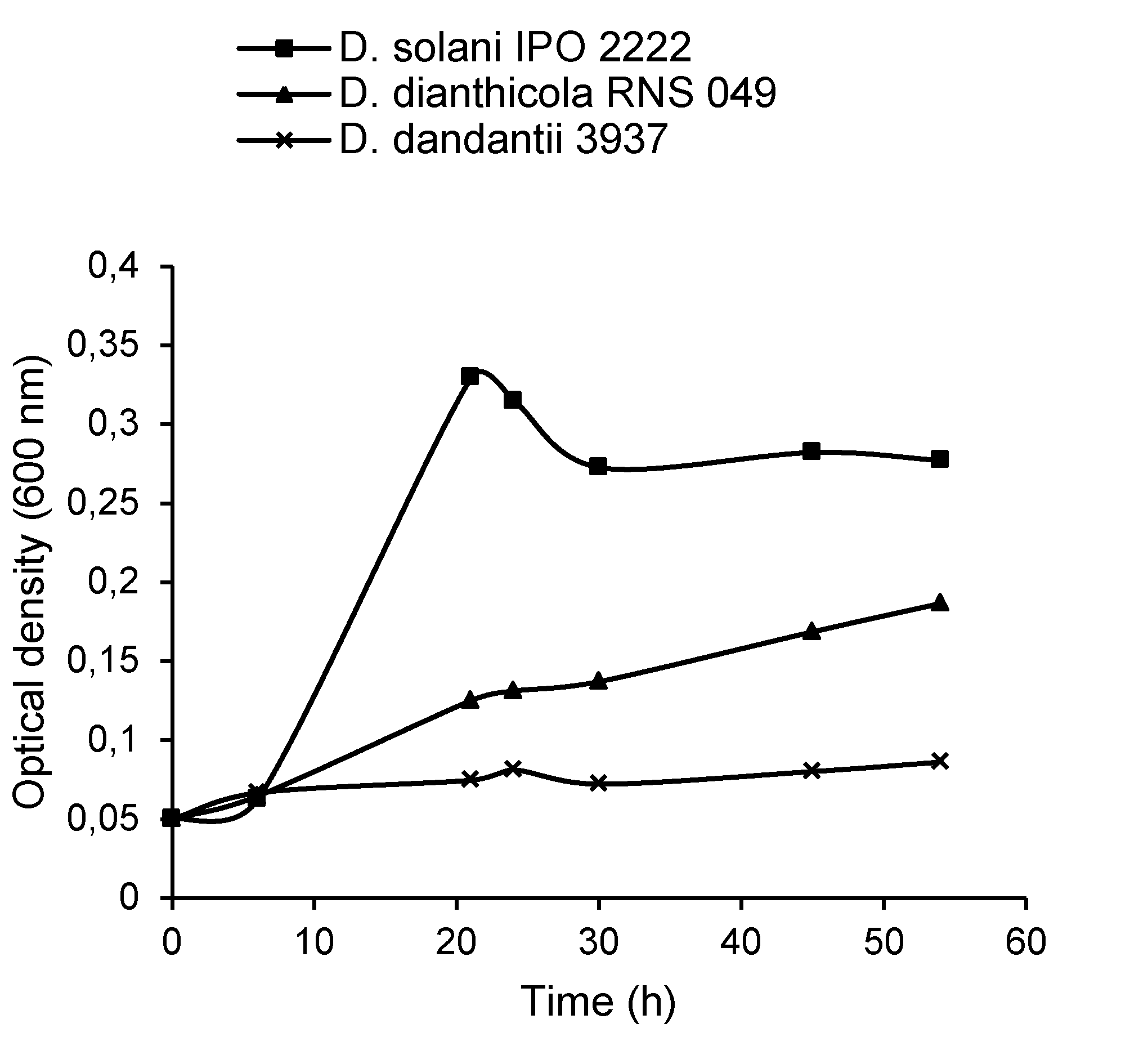

Supplement: Additional file 1: Figure S1. — Growth curves of D. solani IPO 2222T, D. dadantii 3937 and D. dianthicola RNS 049 in the presence of urea as a sole nitrogen source. Data were collected from duplicates. (TIFF 296 kb) [file 40793_2016_208_MOESM1_ESM.tiff]

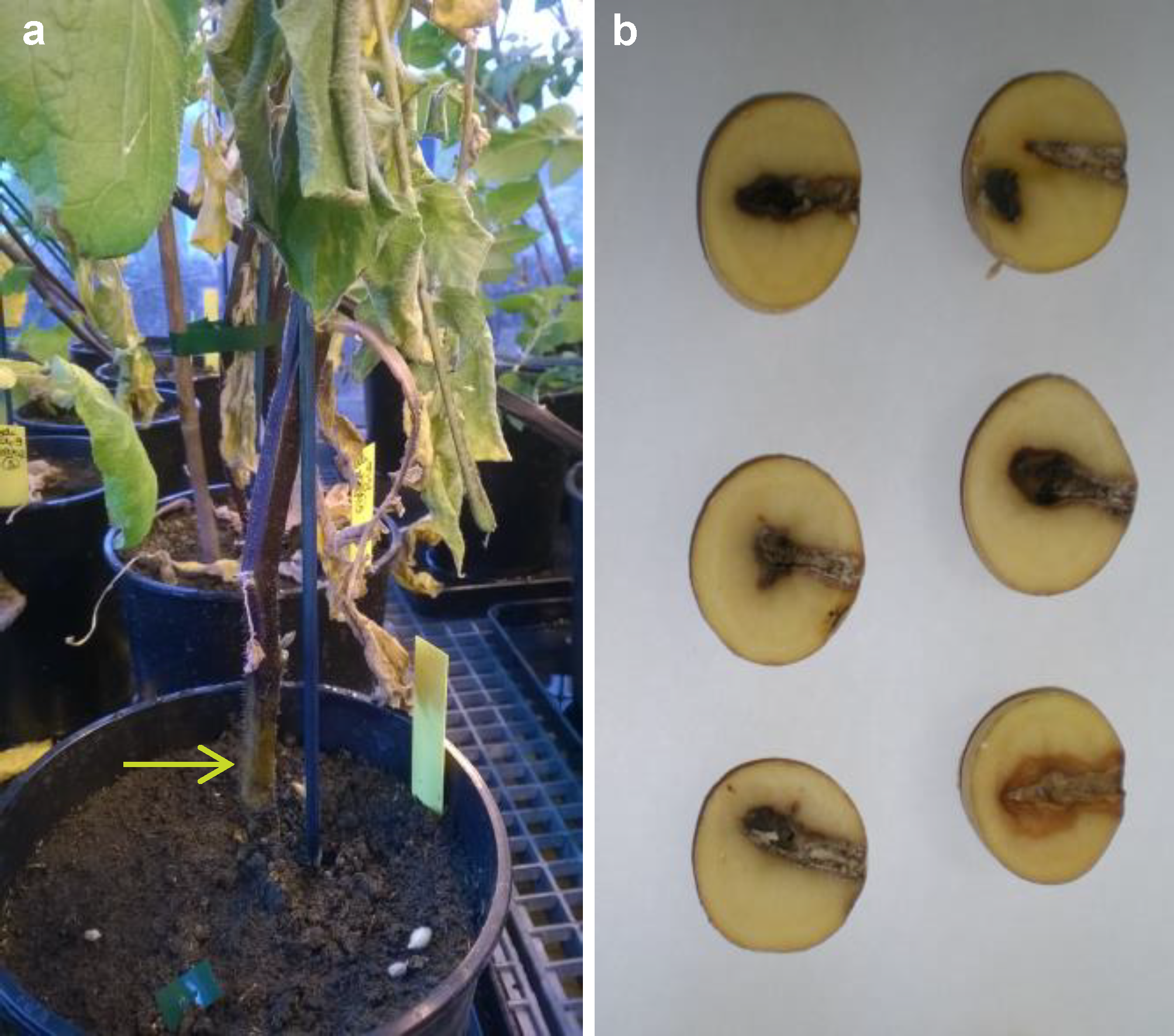

Supplement: Additional file 2: Figure S2. — Symptoms of D. solani IPO 2222T on potato plants (a) and tubers (b). (TIFF 8904 kb) [file 40793_2016_208_MOESM2_ESM.tiff]
